# Acute Care of Patients with Moderate Respiratory Distress: Recommendations from an American College of Emergency Physicians Expert Panel

**DOI:** 10.5811/westjem.43539

**Published:** 2025-09-27

**Authors:** Christopher W. Baugh, Jim F. Neuenschwander, Jesslyn Lenox, Jennifer Hoh, Kara Ward, Sara Muramoto, John Casey, Antonio Anzueto, Hajirah Ishaq, Jared Mount, Peter M. DeBlieux

**Affiliations:** * Brigham and Women’s Hospital, Department of Emergency Medicine, Boston, Massachusetts; † OhioHealth Doctors Hospital, Department of Emergency Medicine, Columbus, Ohio; ‡ South Shore Hospital, Pulmonary Function Lab and Neurodiagnostics, Weymouth, Massachusetts; § University of Louisiana at Monroe, Department of Emergency Medicine, Monroe, Louisiana; ¶ Louisiana State University’s Spirit of Charity Emergency Medicine Residency Program, New Orleans, Louisiana; || Tammany Health System, Division of Pulmonary & Critical Care, St Covington, Louisiana; # Robotic Critical Care Services; ** UT Health San Antonio, South Texas Veterans Health Care System, Division of Pulmonary Medicine/Critical Care, San Antonio, Texas; †† Indiana University School of Medicine, Department of Emergency Medicine, Indianapolis, Indiana; ‡‡ Ohio University Heritage College of Osteopathic Medicine, Department of Medical Education, Athens, Ohio; §§ University Medical Center New Orleans, Department of Emergency Medicine, New Orleans, Louisiana

## Abstract

**Introduction:**

Patients with respiratory distress are frequently encountered in the emergency department (ED). Efforts to assess, initiate treatments, and stabilize these patients require a systematic and rapid response. Emergency physicians need a comprehensive and efficient approach for evaluating, treating, and managing patients presenting to the ED with moderate respiratory distress.

**Methods:**

The American College of Emergency Physicians convened an expert panel of academic and community emergency physicians, critical care specialists, respiratory therapists, hospitalists, and pharmacists to develop and subsequently disseminate consensus recommendations regarding the diagnosis and treatment of patients with moderate respiratory distress presenting to the ED.

**Results:**

A digital tool using a consensus-based framework was developed to aid emergency clinicians in diagnosing and caring for patients with moderate respiratory distress. The tool can be employed at each step in the diagnostic and treatment process.

**Conclusion:**

The evidence-based tool is a practical and freely available bedside instrument for emergency clinicians to diagnose and treat patients with moderate respiratory distress. Further studies are needed to examine the effectiveness of this approach.

## INTRODUCTION

Respiratory distress or shortness of breath represent the third most common reason for visiting the emergency department (ED) in the United States, with 5,918,000 visits in 2021.^1^ Shortness of breath is a common manifestation of a wide array of primary pulmonary and respiratory tract illnesses, as well as other potentially life-threatening conditions, such as acute coronary syndrome, pulmonary embolism, cardiac tamponade, severe anemia, toxic metabolic disturbances, and many others.^2^ Emergency physicians must manage these patients with limited time and information. They also initiate rapid assessment, diagnosis, and treatment to stabilize and begin reversing life-threatening pathophysiology amid increased stressors such as ED boarding and patients with higher morbidity.^3^ Frequent reassessment, adjustments to new data, and observed patient response to therapies are the mainstays of ED care for this patient population.

For emergency physicians faced with the challenge of caring for patients with respiratory distress, many resources, such as specialty society guidelines, are available for disease-specific assessment and treatment.^4^ Few resources are readily available for the initial care of patients with undifferentiated respiratory distress, especially those with moderate symptoms and elevated risk.^5^–^7^ Often, immediate aggressive maneuvers, such as rapid sequence intubation, are not indicated. Developing and disseminating digital tools easily accessed via smartphone or desktop computer, and explicitly designed to support real-time clinical care, is needed.^8^ Lastly, many authoritative guidelines are rarely updated due to rigorous and time-consuming processes for evidence review and consensus generation. This timing results in a delay between the time when innovative and emerging approaches, such as high-flow nasal cannula, are indicated for use and when they are subsequently incorporated into expert recommendations.

Expert panels can be convened to address challenges in clinical practice that students, physicians, nurses, physician assistants, and nurse practitioners may face and provide consensus recommendations. The initial assessment and optimal diagnostic and therapeutic approach to patients with respiratory distress in the ED can be complex and time sensitive. The American College of Emergency Physicians (ACEP) expert panel is a mechanism to address the diagnosis and management of moderate respiratory distress in the ED by providing a practical, consensus-based guide via an online tool and smartphone app at the point of care.

## METHODS

### Study Design

An expert panel to develop a consensus-based decision tool for emergency clinicians was convened by ACEP. Selected panel members conducted a literature review focusing on terms such as “respiratory distress” to examine recent (≤ 5 years) publications and specialty-society guidelines involving the approach to respiratory distress in the ED. The panel sought more recent literature to reflect the current and emerging science related to the target population. When possible, definitions and recommendations reflect study criteria and treatment processes. Otherwise, the expert panel sought to standardize definitions and processes. The panel participated in four virtual rounds of questioning to make recommendations using a modified Delphi rounds process to reach a consensus.^9^ Statements were discussed extensively during the virtual meetings and then serially modified based on discussions. The final expert panel recommendations represented consensus (defined as majority agreement with final adjudication by co-chairs) and majority opinions. This project relied on publicly available sources and was framed as a quality improvement project exempt from institutional review board oversight.

**Population Health Research Capsule**

What do we already know about this issue?

*Patients frequently present to the ED with undifferentiated moderate respiratory distress.*

What was the research question?

*We convened an expert panel and developed a digital tool to assist clinicians’ bedside assessment of respiratory distress in an efficient and evidence-based manner.*

What was the major finding of the study?

*We emphasize a rapid yet thorough initial history and exam to guide diagnosis, initiate targeted treatments, and select the most appropriate disposition for this target patient population.*

How does this improve population health?

*As emergency clinicians face challenges to assess and treat complex patients, digital tools like the one we developed and implemented can help improve care for higher risk populations.*

The definition of moderate respiratory distress is intricate and not easily defined. Our recommendations defined moderate respiratory distress as “a patient who requires more than basic respiratory care or is at risk of deterioration.” For example, this definition would include a patient with a respiratory rate > 24 breaths per minute, with or without hypoxia, and with the use of accessory muscles of respiration.

### Setting

The multidisciplinary expert panel, which was diverse in geography and practice settings, was convened virtually between January–July 2024.

### Selection of Participants

The American College of Emergency Physicians selected the expert panel; the chosen co-chairs Jim F. Neuenschwander and Peter M. DeBlieux then selected additional panelists. Criteria for selection included individuals with direct personal clinical experience in treating patients with respiratory distress in the ED, prior contributions to ACEP expert panels and/or academic contributions, and diverse clinical practice settings within the group. Clinical backgrounds included emergency medicine, critical care, respiratory therapy, pharmacy, and hospital medicine. The final panel consisted of three academic emergency physicians, two community emergency physicians, one ED pharmacist, one respiratory therapist, and one hospitalist, with panelists representing a broad geographic distribution across the US (eg, Southwest, South, Midwest, Northeast).

## RESULTS

The ACEP moderate respiratory distress emergency medicine point of care (emPOC) tool was developed to communicate the main steps in the evaluation, treatment, and disposition of patients presenting to the ED with moderate respiratory distress. The tool is available on the ACEP website and as a smartphone app for Apple and Android devices.

### History and Physical Exam

Care begins with the initial assessment, which includes obtaining a history of present illness and performing a physical examination. The history may be obtained from the patient, emergency medical services, family, or other historians to assist with differential diagnosis, initial testing, and treatment strategy. Table 1a outlines the key elements of the history that should be obtained for patients presenting to the ED with moderate respiratory distress. In parallel with obtaining the patient’s history, a physical examination should begin immediately as the patient is assessed. We outline the key exam elements in Table 1b.

### Testing

After the initial evaluation, the differential diagnosis generated from the encounter will drive a diagnostic strategy. Testing falls into three broad categories: laboratory studies; imaging studies; and other testing. We outline the key diagnostic considerations in Table 2, which may be modified by local resource availability and the evolution of the differential diagnosis as more information is gathered over the patient’s ED course.

### Treatment

Treatments should be targeted at the clinical scene, addressing the underlying pathophysiology driving the patient’s presentation and helping support their work of breathing to avoid clinical decompensation. Initial maneuvers to re-position the patient, suction the airway, or other immediate interventions may be helpful to improve the patient’s work of breathing. Frequent reassessments are a critical aspect of the care of this patient population. The goal is to choose the best intervention to match the patient’s current and anticipated status with frequent evaluation of the need for respiratory support escalation or de-escalation.^10^ As a result, the clinician should proceed with continuous information gathering and optimization of a differential diagnosis, work of breathing, serial vital signs, serial mental status exams, repeat imaging (eg, point-of-care ultrasound), lab result analysis (eg, serial blood gasses), disposition trajectory, evolving goals of care, and delivery of more targeted treatments as the diagnosis becomes clearer.

**Figure 1:**
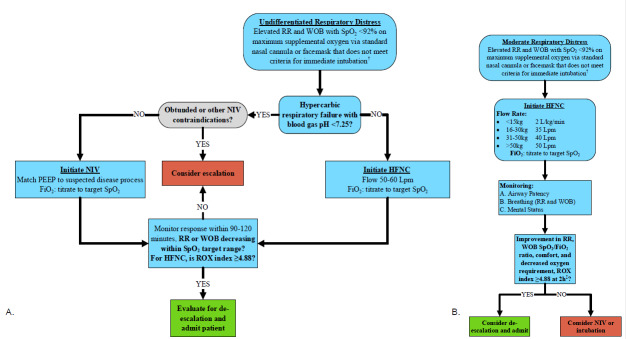
**A.** Adult moderate respiratory distress algorithm. **B.** Pediatric moderate respiratory distress algorithm. ^†^Criteria for immediate intubation include inability to protect the airway, anticipated clinical course, and failure of oxygenation or ventilation despite other treatments (e.g., supplemental oxygen, non-invasive ventilation, or not a candidate for those therapies). ^‡^The extent and reliability of ROX index validation in children remain limited and variable. Patient care algorithm for adult and pediatric patients presenting to the emergency department with moderate respiratory distress. *Lpm*, liters per minute; *RR*, respiratory rate; *NIV*, noninvasive ventilation; *WOB*, work of breathing; *HFNC*, high-flow nasal cannula; *SpO_2_*, peripheral oxygen saturation; *ROX*, ratio of oxygenation; *PEEP*, positive-end expiratory pressure; *FiO_2_*, fraction of inspired oxygen. **Figure 1B adapted from: Mosier JM, Tidswell M, Wang HE. Noninvasive respiratory support in the emergency department: Controversies and state-of-the-art recommendations. *J Am Coll Emerg Physicians Open*. 2024;5(2):e13118. doi:10.1002/emp2.13118 and Kwon JW. High-flow nasal cannula oxygen therapy in children: a clinical review. *Clin Exp Pediatr*. 2020;63(1):3–7. doi:10.3345/kjp.2019.00626.

When using a high-flow nasal cannula, the ratio of oxygenation (ROX) index predicts clinical decompensation and the need for intubation.^11^–^13^ The ROX index is the SpO_2_ (peripheral oxygen saturation)/FIO_2_ (fraction of inspired oxygen) (%) ratio to respiratory rate (breaths/minute). Values ≥ 4.88 measured at 2, 6, or 12 hours after high-flow nasal cannula initiation are associated with a lower intubation risk, whereas values < 3.85 indicate a high risk of high-flow nasal cannula failure and intubation consideration. Intermediate ROX values should be reassessed serially, 1–2 hours later. We illustrate an algorithm for treating and reassessing patients with moderate respiratory distress in Figures 1a and 1b for the adult and pediatric populations, respectively.^14^,^15^

**Figure 2:**
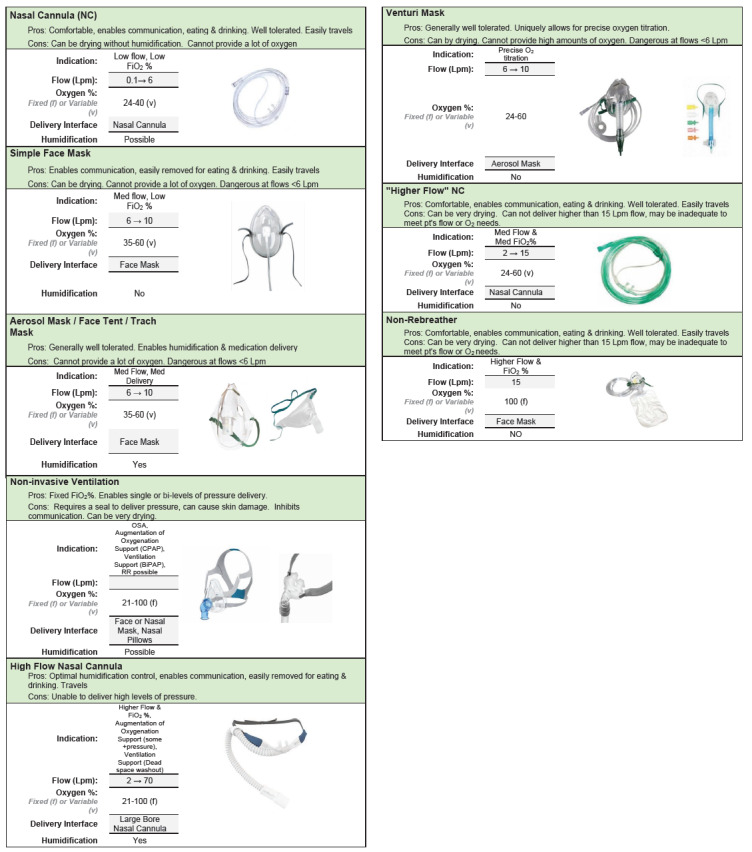
Summary of respiratory therapy options. Additional details for respiratory therapy options for patients presenting to the emergency department with moderate respiratory distress. *BIPAP*, bilevel positive airway pressure*; CPAP*, continuous positive airway pressure*; FiO*_2_, fraction of inspired oxygen; *Lpm*, liters per minute *O*_2_, oxygen; *RR*, respiratory rate, *pt*, patient.

Various respiratory therapies are available in the ED and should match the patient’s needs. Table 3 highlights the key similarities and differences between commonly available respiratory therapies, including nasal cannula, facemask, high-flow nasal cannula, non-invasive ventilation, intubation, and surgical airways. We provide additional detail for each treatment in Figure 2. For some patients, intubation and mechanical ventilation avoidance may be an important consideration, as intubation may increase the risk of peri-intubation complications. These patients may require special ventilation strategies and include examples such as status asthmaticus, severe metabolic acidosis, and untreated pneumothorax.

Medications are an essential component of treatment for most patients in the ED with moderate respiratory distress. Given the broad and variable causes of respiratory distress, we outline key pharmacologic treatments for selected common conditions in Supplement Table 1. This resource is not intended to be exhaustive, and once a specific diagnosis is strongly suspected or confirmed, we recommend that clinicians tailor treatments to best treat specific conditions.

### Disposition

Disposition decisions should be made based on the severity of symptoms, concurrent comorbidities, and the social drivers of health that impact a patient’s ability to adhere to a care plan. Patients from the ED will be discharged home, admitted to the hospital (including inpatient or observation status), or admitted to the intensive care unit. Patients who want to focus on symptom control and quality of life can be directed to palliative care or hospice.

Patients with mild-to-moderate symptoms who respond well to initial therapy in the ED and do not require new or additional supplemental oxygen may be suitable for discharge home. Patients who are being considered for discharge home should meet the following additional requirements: able to ambulate without a new significant drop in oxygen saturation or return of presenting symptoms; able to obtain prescribed medications promptly; able to express an understanding of their medications and how to properly take them, patient or caregiver can perform discharge instruction “teach back” and demonstrate proper use of new and existing therapies; able to express understanding of return precautions (ie, worrisome signs and symptoms that warrant a return to the ED), and able to follow up with an outpatient clinic within 1–2 weeks of the ED visit. Discharge instructions should include a written discharge plan that contains a list of newly prescribed medications, including their purpose and directions (eg, frequency and duration), a list of all home medications, including any adjustments or changes, signs or symptoms that would warrant returning to the ED, and clear directions to follow up with a primary care physician or specialist in a specified time frame. For patients who smoke or live with someone who smokes, smoking cessation information should be given with the discharge paperwork and discussed at the bedside before discharge.

Patients should be hospitalized if they have a new supplemental oxygen requirement. This includes those who have an increased need for supplemental oxygen above their baseline, advanced lung disease, and/or multiple comorbidities, and those who are otherwise unlikely to do well if discharged home. Additionally, those with a new concurrent condition, such as pneumonia, arrhythmias, heart failure, and sepsis, or who have persistent symptoms despite initial ED treatment and require constant monitoring or titration of ventilatory support, should be hospitalized. Finally, those not meeting the criteria for a safe discharge home, including those with social drivers of health likely to disrupt the discharge plan, should remain until a safe plan is identified. Many hospitals have disease-specific protocols that help emergency clinicians determine patient eligibility for observation vs inpatient care, including using ED observation units and home hospital services. We recommend that clinicians reference their local protocols to help direct patients toward the most appropriate site of care in their institution. Example ED observation protocols can be found on ACEP’s Observation Services Toolkit page.

Based on institution-specific capabilities and policies, consider intensive care unit admission for patients with moderate-to-severe symptoms who require escalating respiratory support, are hemodynamically unstable, are intubated, have severe acid-base disturbances, have a new altered mental status, or other symptoms that necessitate close monitoring. Complex patients who may exceed local capabilities or are better served at a higher resource facility require an interfacility transfer. Finally, patients who want to focus on symptom control and quality of life primarily can be directed to palliative care or hospice.

## DISCUSSION

In this paper, we describe our process and consensus recommendations generated by an ACEP-convened expert panel focused on ED care of patients presenting with moderate respiratory distress. Our recommendations emphasize a rapid yet thorough initial history and exam to create and narrow a differential diagnosis, initiate treatments, and select the most appropriate disposition for this target patient population. The framework is presented as a digital tool, available via smartphone app and desktop computer and designed for real-time accessibility to assist clinicians at the bedside for this challenging patient population.

Prior investigations and tools are overwhelmingly targeted at a specific diagnostic tool or therapy for a disease process. For example, evidence abounds for the roles of point-of-care ultrasound and high-flow nasal cannula for the initial evaluation and treatment of patients with undifferentiated respiratory distress.^16^–^19^ Disease-specific guidance for respiratory illnesses such as chronic obstructive pulmonary disease, heart failure, pulmonary embolism, and pneumonia is also available.^4^,^20^–^22^ Alternatively, other resources with a broad approach have not been designed as a tool for real-time clinical support.^2^,^23^ As a result, our novel approach of combining broad patient assessment and treatment guidance with a format optimized for bedside use is novel and designed for maximal clinical impact. This combination best reflects the real-world scenario of the initial ED encounter, where the diagnosis is not yet confirmed, and the support of a digital tool may be most helpful.

The role of electronic clinical decision-support tools is rapidly evolving. Following the framework of the five “rights” of clinical decision-support, our moderate respiratory distress tool aims to provide the right information to the right people in the right channel, format, and time in the workflow.^24^ Evidence is increasing for the use of interactive digital tools at the bedside for high-acuity scenarios, such as pediatric cardiac arrest.^25^ Enabling prompts for clinicians to gather key information and act quickly with evidence-based interventions has the promise of improving care for vulnerable patients, including those with moderate respiratory distress. Future efforts should focus on iterating the content to keep it up to date and improve accessibility, perhaps allowing for electronic health record integration. Such integration may allow for incorporating clinical data feeds (eg, vital signs, laboratory results) and facilitating bedside orders, test result interpretation, and clinical documentation.^26^ While these suggestions have been thoughtful in their creation, we recommend outcome measures in Table 4 that can be used as measures of success.

## LIMITATIONS

Convening an expert panel to develop recommendations and create a bedside tool for emergency physicians has several limitations. First, when evidence was lacking, some aspects of the best practices in the ED were derived only from expert panel discussions. A rigorous systematic literature review and grading consistent with Preferred Reporting Items for Systematic reviews and Meta-Analyses (PRISMA) standards or Grading of Recommendations Assessment, Development, and Evaluation (GRADE) methodology were not performed, and specialty society treatment guidelines formulated via a structured or systematic review of the evidence are typically restricted to interventions with the highest quality of evidence.^27^,^28^ This expert panel’s recommendations are distinct from ACEP clinical policies, governed by the Clinical Policies Committee under a separate process. In addition, outside the relative cost information briefly provided in Table 3, recommendations from this expert panel do not include more detailed economic analyses of the direct and net financial impact of various treatment and disposition (ie, post-ED care) options.

## CONCLUSION

The evidence-based emPOC tool was developed by a multidisciplinary panel of experts convened by ACEP to be a resource for emergency clinicians caring for patients with moderate respiratory distress. Studies examining the functionality of this tool in real-world practice are warranted, as data related to its daily use in the ED setting will allow for improvements and modifications to be rapidly incorporated over time, given the tool’s flexibility to be easily updated. Future studies examining the impact of specific interventions, such as various respiratory therapies initiated during the ED visit vs deferring to the inpatient team, are important to demonstrate the real-world effect of digital tools and further support the benefits of specific interventions on patient outcomes.

## Supplementary Information



## Figures and Tables

**Table 1a:** History elements for patients with moderate respiratory distress.

Dyspnea at rest and with ambulation Chest pain quality, including pleuritic pain Increased sputum production Fever Heart failure Orthopnea Paroxysmal nocturnal dyspnea Cough Peripheral edema Fatigue Altered mental status Weakness Smoking/vaping use Chronic lung disease (e.g., chronic obstructive pulmonary disease, interstitial lung disease, pulmonary fibrosis) Anemia Asthma Increased sputum/color/production/volume Current oxygen requirement Prior intubation or known difficult airway Recent travel (e.g., elevation, exposure, pulmonary embolism) Known infectious contacts Prior personal or family history of venous thromboembolism Terminal diagnosis/condition Exogenous estrogen use Hemoptysis Hospitalizations Trauma/surgery (e.g., fat emboli, rib fractures, pulmonary contusion, flail chest) Dysphagia/severe reflux Environmental exposure (e.g., carbon monoxide, asbestos) Congenital anomalies (e.g., Marfan, cystic fibrosis, Ehlers-Danlos) Cancer Inflammatory (e.g., lupus, rheumatoid arthritis) Substance use disorder Goals of care Pain Anxiety Immunosuppressed state

**Table 1b:** Physical examination elements for the patient with moderate respiratory distress.

Vitals trending, including prehospital Tachypnea Tachycardia Hypotension Upper airway exam Voice quality Abnormal lung exam (e.g., wheezing, rhonchi, crackles) Hypoxia Tripoding or accessory muscle use Mental status Abnormal skin exam (e.g., pallor or discoloration, urticarial rash) Swallow Cough quality Grunting, nasal flaring, or abdominal contractions Diaphoresis Irregular breathing pattern Crepitus Extremity edema Screening for difficult airway assessment and management: (https://www.aliem.com/mnemonics-for-difficult-airway/)

History and physical exam elements for patients presenting to the ED with moderate respiratory distress.

**Table 2:** Diagnostic approach to the patient with moderate respiratory distress.

General Labs
Complete blood count (CBC)
anemia, pneumonia/infections
Comprehensive metabolic panel (CMP)
ketoacidosis (diabetic ketoacidosis [DKA]/starvation ketoacidosis [SKA]/alcoholic ketoacidosis [AKA]), pulmonary edema (cardiorenal effects), electrolytes (cardiac arrhythmia), metabolic acidosis
Point of care blood gas
Targeted Labs
Blood gas: use pulse ox and bicarb on basic metabolic panel (BMP)
Co-oximetry
carbon monoxide, methemoglobinemia
Venous blood gas (VBG)
chronic obstructive pulmonary disease (COPD), asthma, respiratory failure
Brain natriuretic peptide (BNP)
heart failure, cardiomyopathy, acute coronary syndrome (ACS)
International normalized ratio (INR) with a history of anticoagulation or coagulopathies
Anti-factor Xa test if actively bleeding
Troponin
acute coronary syndrome (ACS)/pulmonary embolism (PE)/venous thromboembolism (VTE)
D-dimer
PE rule out
Lactate
seizure, sepsis
Procalcitonin
pneumonia or other infection
Blood cultures
Nasal swab for viral panel
C-reactive protein (CRP)
chronic lung disease
Toxicology
salicylate
alcohol and urine tox
Targeted imaging/studies
Chest x-ray (CXR)
pneumonia, pleural effusion, enlarged cardiac silhouette, widened mediastinum, pneumomediastinum, rib fractures, pneumothorax, subcutaneous air, acute respiratory distress syndrome (ARDS), mass, atelectasis, small inspiratory lung volumes (neuromuscular disease)
Expiratory films for focal air trapping/unilateral wheezing
foreign body, mucous plug
Computed tomography chest
with contrast
concern for mediastinal mass or pleural metastases
without contrast
pneumonia, ground glass opacities, eval pulmonary
nodule/mass seen on x-ray, pulmonary contusions,
ARDS, pulmonary edema, rib fractures
CT angiogram (CTA) Chest
PE aortic dissection, trauma
Ventilation/Perfusion (V/Q) scan - hard to interpret with existing pulmonary disease/abnormal CXR
PE
Point of care ultrasound
bedside: pneumonia, pneumothorax, pleural or pericardial effusion, pulmonary edema, cardiac tamponade, right heart strain (PE, right heart failure), left ventricular (LV) function, ability to tolerate fluids, inferior vena cava (IVC), lung windows
Consider Ancillary Testing:
End-tidal CO_2_ (ETCO_2_)
waveform analysis for overdose, asthma, and COPD
Electrocardiogram (EKG)
arrhythmia, ACS
Negative inspiratory force (NIF)
neuromuscular disease
Physical exam maneuvers
fluid status
passive leg raise-fluid responsiveness

Diagnostic testing considerations for patients presenting to the emergency department with moderate respiratory distress.

*ACS*, acute coronary syndrome; *CO_2_*, carbon dioxide; *COPD*, chronic obstructive pulmonary disease.

**Table 3:** Comparison of respiratory therapies.

NC, mask, NRB	HFNC	NIV	ETT/Surgical neck airway
FiO_2_ estimated	FiO_2_ controlled		
Well-tolerated	Tolerated	Uncomfortable	Medicated
Inexpensive, simple	Intermediate cost, Increased resource intensity	Expensive, resource intensive	
No PEEP, No Pressure Support	PEEP (Low level)	PEEP +/− Pressure Support	
Cooperation Not Needed		Cooperation Needed†	Not Needed
Patient clears secretions		Assisted secretion clearance	

† Patient participation is required.

Respiratory therapy options for patients presenting to the emergency department with moderate respiratory distress.

*NC*, nasal cannula; *Mask*, facemask; *NRB*, nonrebreather; *HFNC*, high-flow nasal cannula; *NIV*, non-invasive ventilation; *ETT*; endotracheal tube; *FiO*_2_, fraction of inspired oxygen; *PEEP*, positive end-expiratory pressure.

** Adapted from Baker K, Greaves T, Fraser JF. How to use humidified high-flow nasal cannula in breathless adults in the emergency department. *Emerg Med Australas*. 2019 Oct;31(5):863–868. doi: 10.1111/1742-6723.13372. Epub 2019 Aug 6. PMID: 31389171.

**TABLE 4:** Suggested outcome measures for patients with moderate respiratory distress.

Emergency department door-to-disposition decision times Intensive care unit admission volume and rate Hospital length of stay Therapy switches (i.e., high flow nasal cannula to NIPPV) and escalations to intubation 30-day emergency department revisits and hospital readmissions

*NIPPV*, non-invasive positive pressure ventilation.
