# Supplementary material for: Acute Care of Patients with Moderate Respiratory Distress: Recommendations from an American College of Emergency Physicians Expert Panel
Source: West J Emerg Med. 2025 Sep 27;26(5):1485–94. doi: 10.5811/westjem.43539 (PMC12591642; doi:10.5811/westjem.43539)
Supplement: Supplementary file 1 [file wjem-26-1485-s001.docx]

**Supplement Table 1.** Targeted medications for selected conditions.

**Medications for Consideration**

- Two scenarios:

1. Patient remains undifferentiated → next steps, disposition endpoints
2. Most likely diagnosis confirmed → enter disease-specific treatment algorithm (e.g., COPD, asthma, congestive heart failure [CHF], pneumonia, anaphylaxis, PE, etc.)

**Signs of wheezing or decreased air movement**

- Bronchodilators (short-acting beta2 agonists)
  - Albuterol
    - Via nebulization: 2.5 to 5 mg per nebulized treatment every 10-20 minutes for the first hour. Can also consider up to 20 mg per hour for continuous nebulization
    - Via metered dose inhaler with spacer: up to 4 puffs (90 mcg/puff), can increase to 8 inhalations for patients requiring mechanical ventilation up to every hour for initial management

**Signs of airway inflammation**

- Consider systemic corticosteroid administration
- Intravenous therapies may include methylprednisolone **or** dexamethasone

**Signs of upper airway obstruction (considering anaphylactic reaction as a cause)**

**Epinephrine**

- Consider first line for severe anaphylactic reactions; alpha and beta agonists to decrease angioedema, reverse vasodilation, and bronchoconstriction
- Adult dosing: 0.3-0.5 mg IM into the lateral thigh, can repeat every 5-15 minutes for two doses
- Epinephrine should be administered using the 1 mg/mL concentration for anaphylaxis/IM administration
- If unresponsive after two doses, consider initiation of an IV epinephrine infusion starting at 1 mcg/minute and increasing 1 mcg/minute every 5 to 10 minutes for a maximum dose of 10 mcg/minute
- Additional therapies such as antihistamines and systemic corticosteroids may be considered in patients presenting with non-respiratory manifestations
- These agents may be considered as second line in patients presenting with respiratory manifestations

**Signs of fluid overload (in patients with heart failure)**

- Nitroglycerin
  - Consider reducing afterload via arterial dilation in the setting of acute pulmonary edema. This can be considered as an adjuvant to diuretic therapy.
  - Adult (high) dosing: up to 100 mcg/minute
  - Higher initial doses of nitroglycerin may be effective in shortening time to symptom resolution, achieving blood pressure targets, and decreasing intubation rates
  - Use with caution in patients with hypotension as nitroglycerin decreases blood pressure
- Loop Diuretics
  - Can be considered to relieve congestion, improve symptoms and prevent worsening of HF exacerbation. Loop diuretics work to inhibit the reabsorption of sodium and chloride in the ascending loop of Henle.
  - Furosemide
  - Adult dosing: initiate IV therapy with at least two times the daily home (oral) diuretic dose
  - IV to PO conversion is 1:2 (ie, furosemide 20 mg IV is equivalent to 40 mg PO)
  - IV doses > 120 mg should be given as an IV infusion to prevent ototoxicity
  - Furosemide 20 mg IV = Torsemide 20 mg IV = Bumetanide 1mg

**Signs of leg swelling, tachycardia (concern for a pulmonary embolism)**

- Pulmonary embolism treatment strategies are guided by a risk-adjusted management strategy. Reperfusion treatment is recommended for high-risk patients (hemodynamic instability) and intermediate-high risk (hemodynamically stable, troponin positive, and right ventricular dysfunction).
- For low-risk PE treatment, refer to the [Low-risk PE Point of Care Tool](https://poctools.acep.org/POCTool/Low-riskPE/2b7ac2d5-45ee-429f-9e60-42e3c77dfc94/)
- Alteplase
- Consider use in patients who are high risk (eg, hemodynamic instability) with PE confirmed or strong clinician suspicion (ie, hemodynamic instability with PE risk factors)
- Alteplase is a fibrinolytic agent that induces fibrinolysis through the conversion of plasminogen to plasmin
- Adult dose: 100 mg IV over 2 hours
- Low molecular weight heparin
- Consider use in patients who are intermediate-high risk and intermediate-low risk
- Adult dose: 1 mg/kg every 12 hours or 1.5 mg/kg every 24 hours
- Unfractionated heparin
- Consider use in patients who are intermediate-high risk and intermediate-low risk
- Adult dose: 80 units/kg IV bolus followed by a continuous IV infusion starting at 18 units/kg/hr

**Signs of fever, cough, sputum production (concern for pulmonary infection)**

- Prompt initiation of an empiric, broad-spectrum antibiotic regimen is recommended
- If sepsis is suspected, follow institutional sepsis protocols

**Asthma Exacerbations -** Refer to the [Asthma Point of Care Tool](https://poctools.acep.org/POCTool/AsthmaExacerbation/29dd238b-2b23-41e1-9baa-6cb27e6056c0/) for more detailed information. Medications to consider include:

- Short-acting beta2 agonists
- Short-acting antimuscarinics (anticholinergics)
- Systemic corticosteroids
- Magnesium

**COPD Exacerbations** - Medications to consider include:

- Short-acting beta2 agonists
- Short-acting antimuscarinics (anticholinergics)
- Systemic corticosteroids
- Antibiotics

Medication treatment options for patients presenting to the emergency department with moderate respiratory distress.

*COPD*, chronic obstructive pulmonary disease; *IV to PO*, intravenous to oral; *PE*, pulmonary embolism.
